# Retraction: Barriers and facilitators of childhood obesity prevention policies: a systematic review and meta-synthesis

**DOI:** 10.3389/fped.2024.1390512

**Published:** 2024-02-29

**Authors:** Frontiers Editorial Office

A Retraction of the Original Research Article Barriers and facilitators of childhood obesity prevention policies: a systematic review and meta-synthesis By Taghizadeh S, Hashemi MG, Zarnag RK, Fayyazishishavan E, Gholami M, Farhangi MA and Gojani LJ (2023). Front. Pediatr. 10:1054133. doi: 10.3389/fped.2022.1054133

The Journal retracts the 2022 article cited above.

Following publication, concerns were raised regarding the contributions of the authors of the article. Our investigation, conducted in accordance with Frontiers policies, confirmed a serious breach of our authorship policies and of publication ethics; the article is therefore retracted.

This retraction was approved by the Chief Executive Editor of Frontiers. The authors received a communication regarding the retraction and had a chance to respond. This communication is recorded by the publisher.

